# The aggressiveness of neurotrauma practitioners and the influence of the IMPACT prognostic calculator

**DOI:** 10.1371/journal.pone.0183552

**Published:** 2017-08-23

**Authors:** Joshua Letsinger, Casey Rommel, Ryan Hirschi, Raminder Nirula, Gregory W. J. Hawryluk

**Affiliations:** 1 Department of Neurosurgery, Clinical Neurosciences Center, University of Utah, Salt Lake City, Utah, United States of America; 2 Department of Biomedical Informatics, School of Medicine, University of Utah, Salt Lake City, Utah, United States of America; 3 School of Medicine, University of Utah, Salt Lake City, Utah, United States of America; 4 Department of Surgery, University of Utah, Salt Lake City, Utah, United States of America; Leids Universitair Medisch Centum, NETHERLANDS

## Abstract

Published guidelines have helped to standardize the care of patients with traumatic brain injury; however, there remains substantial variation in the decision to pursue or withhold aggressive care. The International Mission for Prognosis and Analysis of Clinical Trials in TBI (IMPACT) prognostic calculator offers the opportunity to study and decrease variability in physician aggressiveness. The authors wish to understand how IMPACT’s prognostic calculations currently influence patient care and to better understand physician aggressiveness. The authors conducted an anonymous international, multidisciplinary survey of practitioners who provide care to patients with traumatic brain injury. Questions were designed to determine current use rates of the IMPACT prognostic calculator and thresholds of age and risk for death or poor outcome that might cause practitioners to consider withholding aggressive care. Correlations between physician aggressiveness, putative predictors of aggressiveness, and demographics were examined. One hundred fifty-four responses were received, half of which were from physicians who were familiar with the IMPACT calculator. The most frequent use of the calculator was to improve communication with patients and their families. On average, respondents indicated that in patients older than 76 years or those with a >85% chance of death or poor outcome it might be reasonable to pursue non-aggressive care. These thresholds were robust and were not influenced by provider or institutional characteristics. This study demonstrates the need to educate physicians about the IMPACT prognostic calculator. The consensus values for age and prognosis identified in our study may be explored in future studies aimed at reducing variability in physician aggressiveness and should not serve as a basis for withdrawing care.

## Introduction

Advancement in traumatic brain injury (TBI) care has lagged behind that in other fields [1, 2]. Unfortunately, we remain without a pharmacologic agent that improves outcome from TBI, although progress is being made in elucidating management strategies that benefit patients with TBI. In particular, the development of evidence-based management guidelines—first published in 1996 [3]—have helped to improve and standardize TBI care. Adherence to published guidelines has been associated with improved outcomes [4]. The development and extensive validation of the International Mission for Prognosis and Analysis of Clinical Trials in TBI (IMPACT) prognostic calculator (http://www.tbi-impact.org/?p=impact/calc) has also been an important advance [5]. According to external validation, it accurately predicts outcomes for TBI patients in >80% of cases.

A very important aspect of TBI care that has been the subject of little study is the decision about when to provide aggressive care and when to pursue comfort care or withdrawal of care. In our experience, substantial variation exists in practitioner perceptions of patient outcomes [6] as well as their attitudes and decisions with regards to the pursuit of aggressive care. Of particular concern is when nihilism robs a patient of a reasonable chance of a good outcome. Here, the IMPACT prognostic calculator offers the opportunity to bring greater objectivity to level-of-care decisions and the possibility of greater standardization in provider aggressiveness.

Practitioner familiarity with the IMPACT calculator is unknown [7, 8]. With this in mind, we performed an international, multidisciplinary survey of practitioners who provide acute care to TBI patients with the goal of understanding familiarity with and current uses of the IMPACT prognostic calculator. We sought to determine whether there is a consensus level at which the chance of death or a poor outcome would cause physicians to seriously consider nonaggressive care. Moreover, given that age is the strongest predictor of outcome from TBI [9], we sought to determine if there is a consensus age at which nonaggressive care is strongly considered by practitioners. We additionally explored physician and institutional predictors of physician aggressiveness with the goal of better understanding provider decisions.

## Materials and methods

### Surveys

We conducted a survey of neurosurgeons and other practitioners who provide care to TBI patients in the acute phase (S1 Survey). The American Association for the Surgery of Trauma (AAST) distributed the survey to its members, who are predominantly acute care surgeons, surgical intensivists, and general surgeons. The University of Utah Institutional Review Board serves to ensure protection of human research subjects in research completed by University of Utah investigators. An anonymous survey of physician opinion is exempt from requiring approval of our institutional review board as it poses no risk to the unidentifiable, anonymous respondents who completed our survey. Voluntary completion of the survey was considered equivalent to provision of informed consent.

The 19-question survey we developed was administered by email in October of 2015. The questions were developed to understand current familiarity with and use of the IMPACT prognostic calculator and to investigate how this was influenced by respondents’ experience and practice environments. Questions also explored threshold values of age as well as chance of death or poor outcome that would prompt a serious consideration of withdrawal of care. Survey responses were collected and managed using the Research Electronic Data Capture application (REDCap, Nashville, Tennessee) [10]. We accepted responses during a three-month period (October-December 2015); non-responders were provided with two reminders during this period.

### Statistical analysis

Responses were exported from REDCap electronic data capture tool [10]. Statistical analysis was performed using SPSS version 23 (International Business Machines Corporation, Armonk, New York) and STATA version 12 (StataCorp LP, College Station, Texas). Poisson regression was used for count data; analysis of variance and chi-squared tests were used to analyze means. A Bonferroni correction was done for multiple comparisons. Binomial logistic regression was used for discrete choice data and to determine associations with other features. Error bars represent standard error in all cases.

## Results

### Characteristics of respondents and their institutions

**Fig 1:**
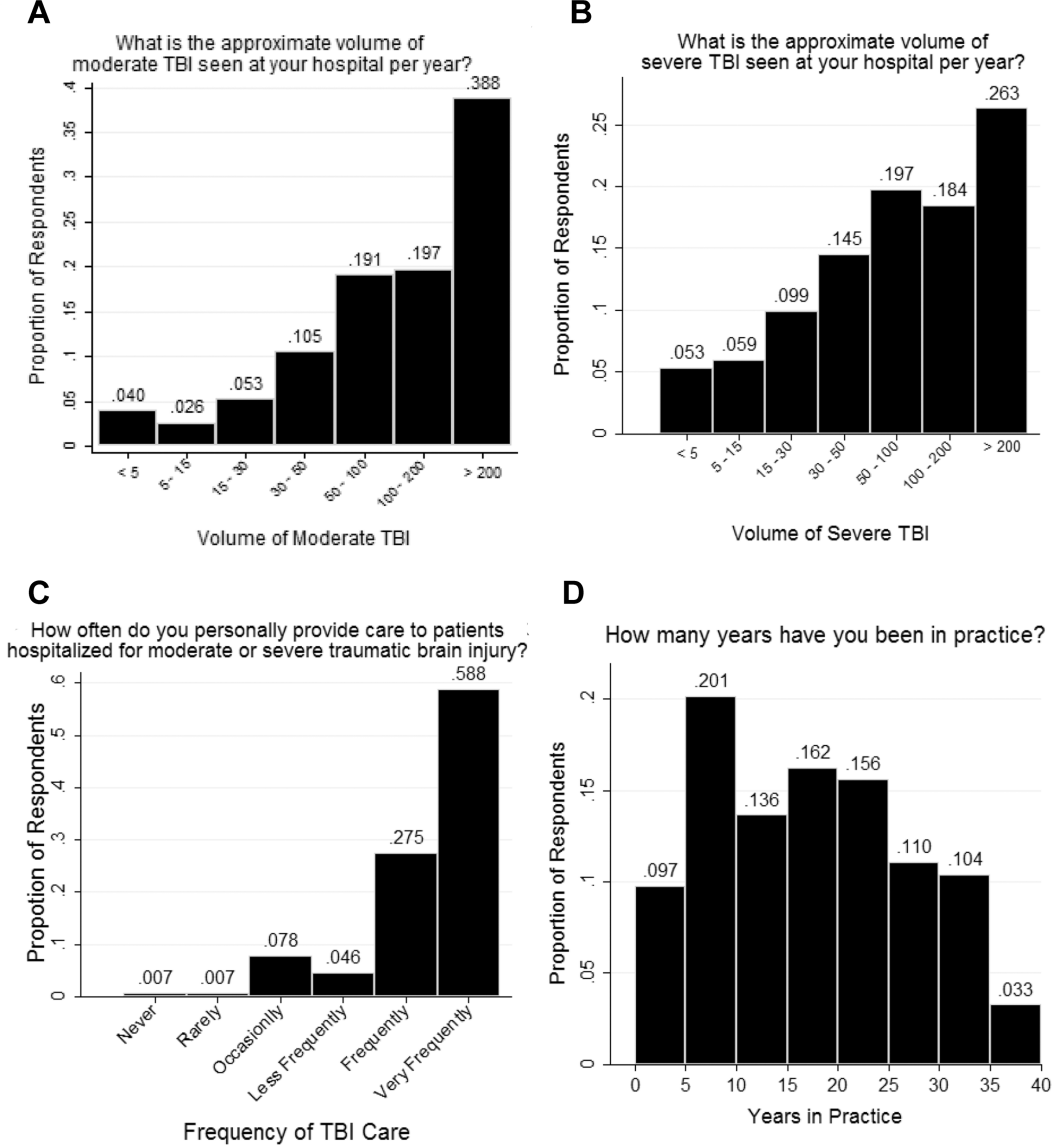
**Responses to survey questions 7, 6, 8, and 2 (S1 Survey, Supplemental Digital Content 1) are presented in A, B, C, and D, respectively. A)** Reported volume of moderate TBI cared for at the respondents’ institutions per year. **B)** Reported volume of severe TBIs cared for at the respondents’ institutions per year. **C)** Reported frequency of individual neurotrauma physicians providing TBI care. **D)** Years in practice for survey respondents.

A total of 154 responses to our survey were received and analyzed. Characteristics of respondents are shown in Table 1. A majority of respondents were general surgeons (71.4%) followed by neurosurgeons (23.4%). A minority were practitioners of other subspecialties who provide acute TBI care (5.2%). Most respondents (80.5%) work at a Level I trauma center, whereas 14.9% work at a Level II trauma center and 4.5% at a Level III center. The volume of moderate and severe TBI reportedly treated by respondents is demonstrated in Fig 1. A majority (58.8%) of the respondents said that they provide care for moderate to severe TBI “very frequently” and 27.5% said that they provide care “frequently” (Fig 1C). Respondents who provide care “less frequently,” “occasionally,” “rarely,” and “never” made up the remaining 13.7% of the cohort. Just over half (54.6%) of the respondents work for institutions with separate surgical critical care and neurocritical care units, whereas the remaining institutions had a single critical care unit providing care to the acutely brain injured. Respondents reported being in practice a mean of 16.5 years (median 15, range 1–40) (Fig 1D).

**Table 1:** Characteristics of survey respondents.

Characteristic	Proportion of Respondents (%)
Neurosurgeons	23.4
General Surgeons	71.4
Other	5.2
Practicing in America	83.1
Practicing Outside America	9.1
Did Not Answer	7.8

### Familiarity with and use of the IMPACT prognostic calculator

**Fig 2:**
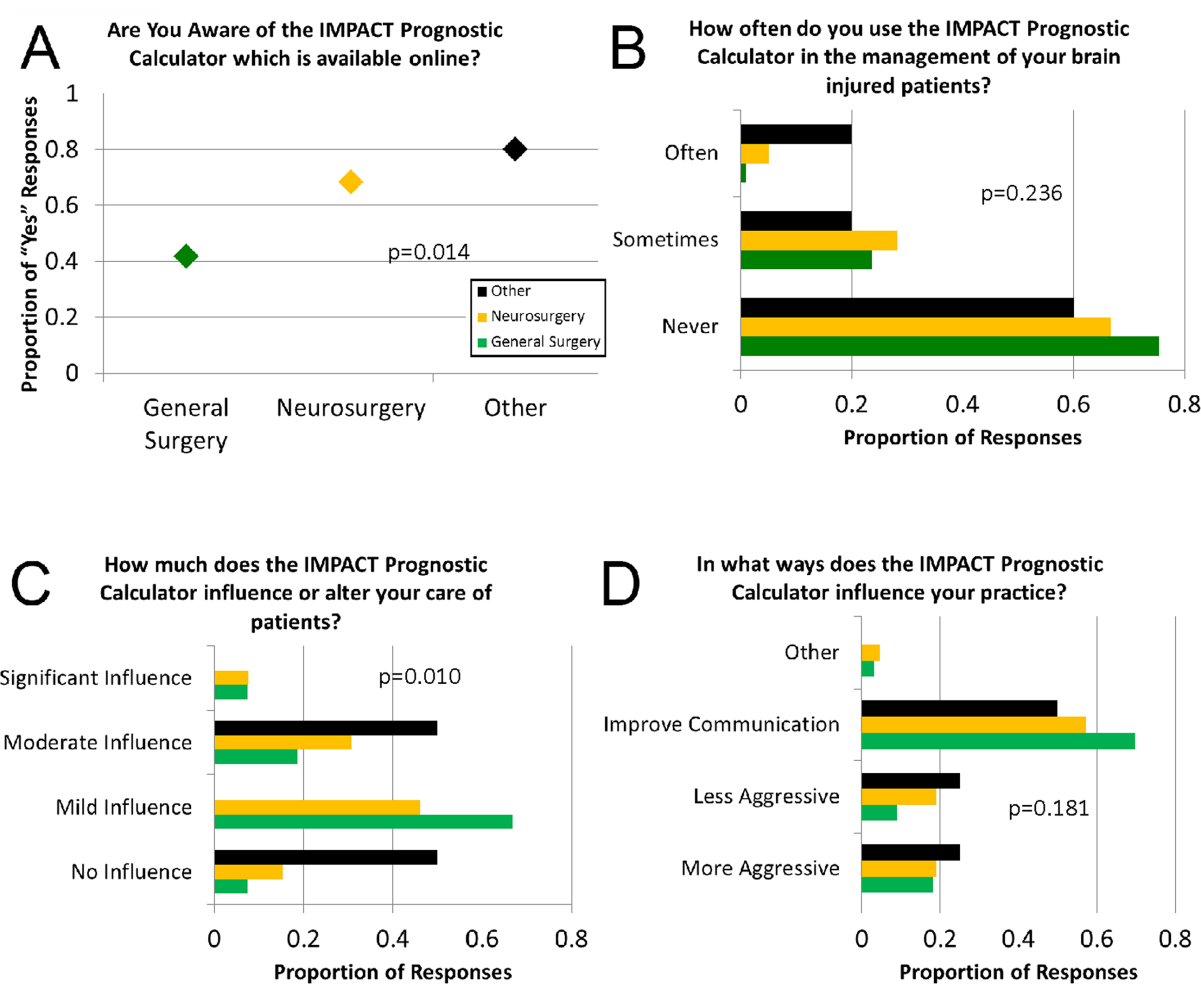
**Responses to survey questions 12, 13, 14, and 15 (S1 Survey, Supplemental Digital Content 1) are presented in A, B, C, and D, respectively.** General surgeons returned a larger number of survey responses (109), neurosurgeons returned 37 responses; the remaining practitioners (neither GS nor NS) returned 7 surveys. **A)** The proportion of respondents that reported awareness of the IMPACT prognostic calculator by specialty. **B)** Reported frequency of use of the IMPACT prognostic calculator in patient management by specialty (p = 0.236). Over 45% of all respondents that were aware of the calculator “Never” use it in practice. **C)** Influence of IMPACT prognostic calculations on patient care by specialty. **D)** Reported uses of the IMPACT prognostic calculator by specialty. Respondents could select more than one option.

Approximately half of surveyed providers (50.3%) answered that they were aware of the online IMPACT calculator. Practice specialty was significantly associated with awareness of the IMPACT calculator, with 69.4% of neurosurgeons reporting awareness of the calculator as compared with 42.2% of general surgeons (p = 0.014, Fig 2A). Among those aware of the calculator, 45.5% said that they never use it in the management of brain-injured patients, 5.2% said that they often use it, and 48.1% said that they sometimes use it (p = 0.236, Fig 2B).

Of 42 providers who at least occasionally used the calculator, 88.1% stated that it had some influence on their care of patients (57.1% said that it had a “mild influence” on their care of patients, 23.8% “moderate influence,” and 7.1% significant influence) (Fig 2C). All of those respondents reporting that the IMPACT calculator had “significant influence” on care (7.1%) had fewer than 10 years in practice. Neurosurgeons reported significantly more often (33.3%) that the IMPACT calculator had a “moderate influence” on their care of patients, whereas only 18.5% of general surgeons responded saying that the calculator had the same influence (p = 0.010, Fig 2C).

For those who were influenced by the calculator, a large majority (91.9%) stated that it helped improve communication about prognosis with family, 21.6% of physicians said that it led them to pursue more aggressive care, and 27.0% indicated they proceeded more quickly to a lower level of care (respondents could select more than one option, Fig 2D). Over 20% stated that the IMPACT predictions influenced care in more than one way. Binomial logistic regression demonstrated that specialty was not significantly associated with the way IMPACT predictions influenced care (p = 0.181, Fig 2D).

### Prognostic calculation and physician aggressiveness

**Fig 3:**
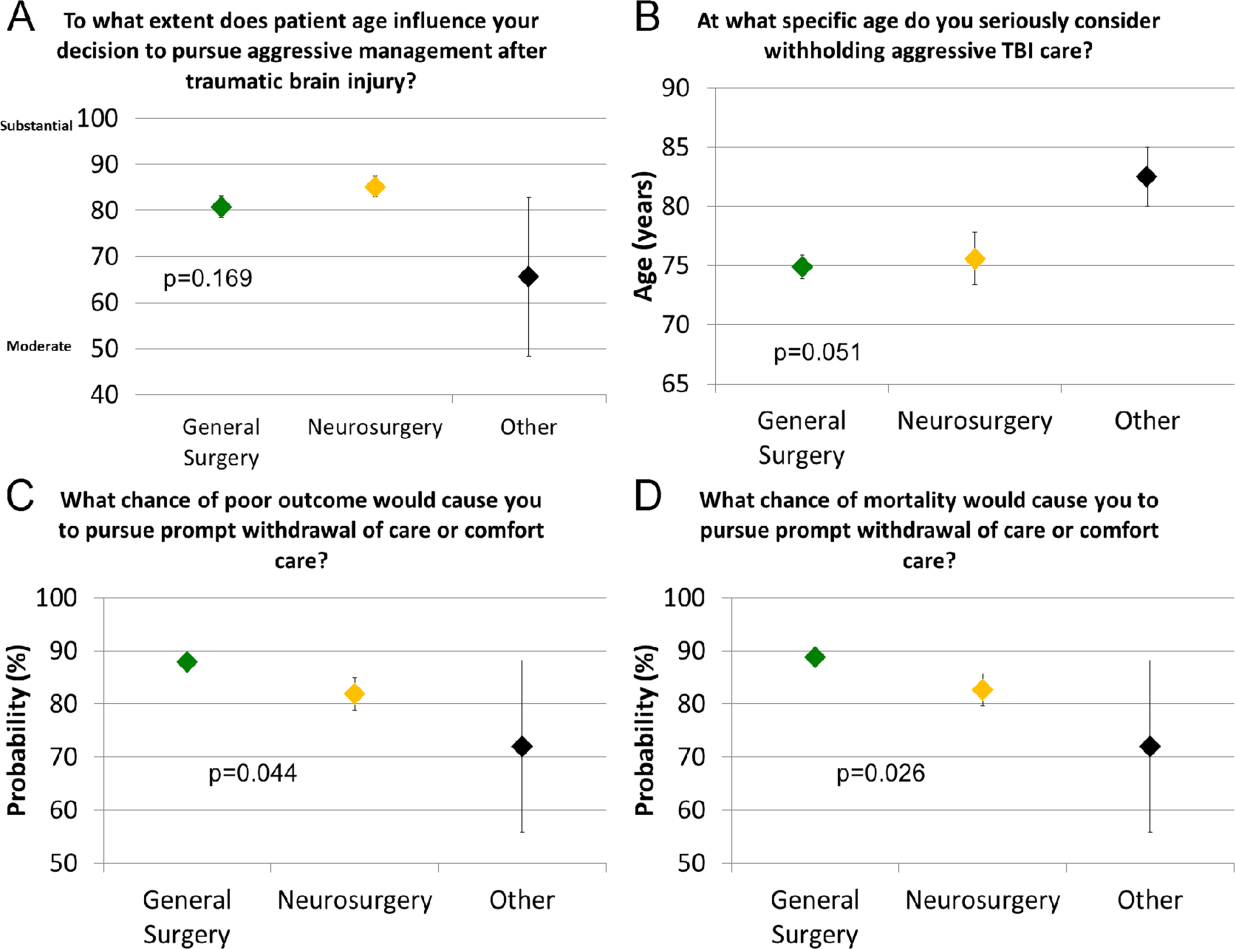
**Responses to survey questions 18, 19, 17, and 16 (S1 Survey, Supplemental Digital Content 1) are presented in A, B, C, and D, respectively.** In our survey, we posed a series of questions that assumed the calculator produced results that were 100% accurate. **A)** Reported extent to which age influences aggressive care among different specialties, by specialty. Just over 10% of all respondents said that age had no influence on their decision making. **B)** Reported age threshold at which one might consider withholding aggressive care, by specialty. **C)** Reported chance of poor outcome that prompts comfort care considerations, by specialty. **D)** Reported chance of mortality that prompts comfort care considerations, by specialty. Error bars represent standard error.

Only 16.7% of neurosurgeons and 14.0% of general surgeons indicated that age has no influence on their decision to pursue aggressive care, and typically respondents indicated that patient age has a strong influence on their decision. On a scale from 0 to 100 with 0 being no influence of age and 100 being maximal influence of age, neurosurgeons responded 85.0 on average while general surgeons responded 81.6 and other practitioners responded 61.1 (p = 0.169, Fig 3A). Considering the average response of all who answered the survey, an age of 75.7 years was favored as an age at which nonaggressive care might be strongly considered. Among neurosurgeons, the average age identified was 77.2 years, for general surgeons it was 74.9 years, and among other practitioners it was 83.3 years (p = 0.051, Fig 3B).

We also sought to determine whether there was a level at which the chance of mortality or poor outcome would prompt practitioners to strongly consider withholding aggressive care. Overall, an 85.9% chance of poor outcome was the average threshold that would prompt the pursuit of comfort care (neurosurgeons 81.4%, general surgeons 87.9%, other practitioners 79.3%) (p = 0.044, Fig 3C). Similar responses were provided with respect to chance of mortality that would prompt consideration of comfort care. Overall, 86.7% chance of mortality was the average that would prompt the pursuit of comfort care (neurosurgeons 82.2%, general surgeons 88.8%, other practitioners 78.6%) (p = 0.026, Fig 3D).

### Predictors of physician aggressiveness

**Fig 4:**
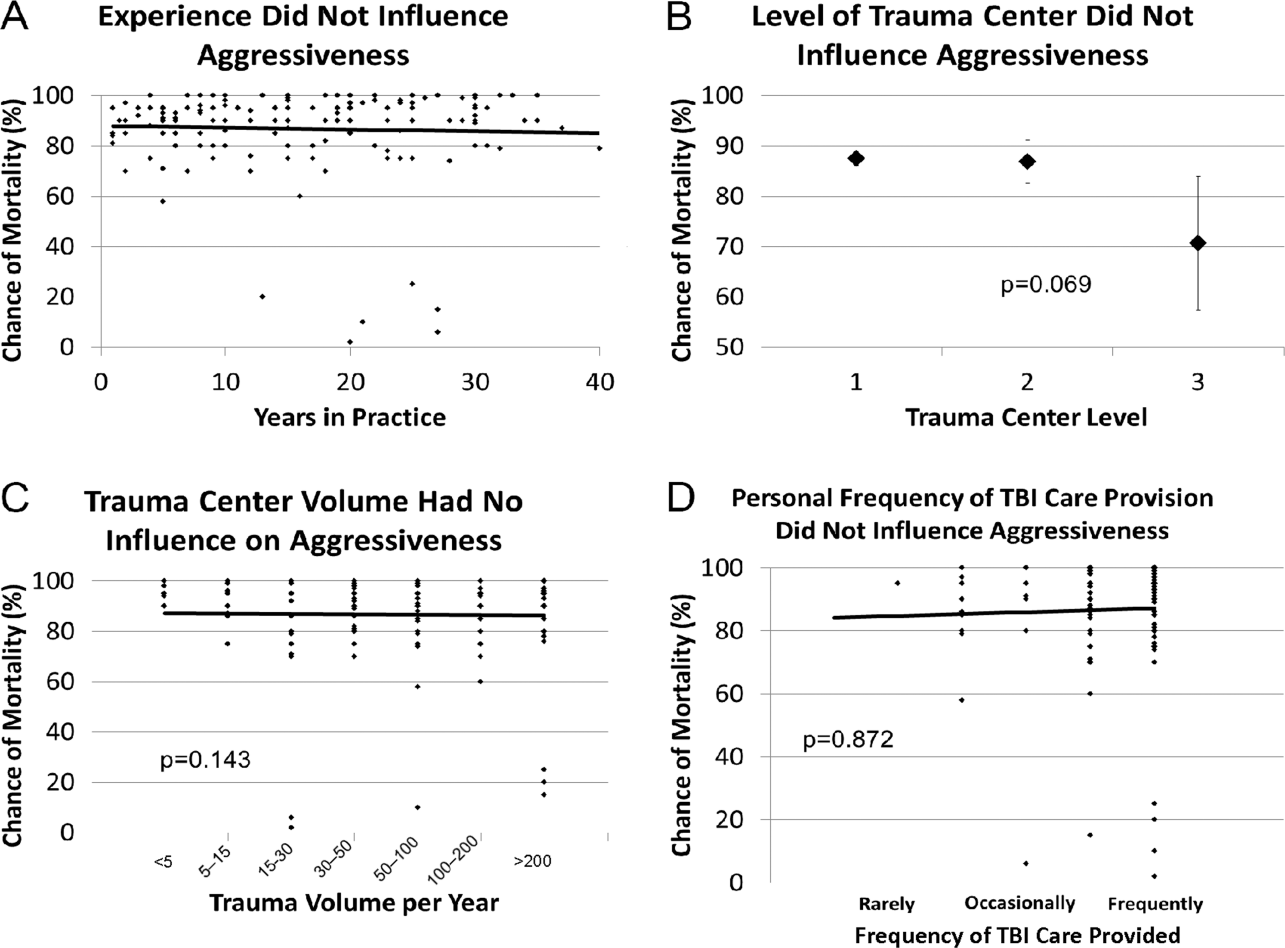
Effect of variables on physician aggressiveness. **A)** Chance of mortality that prompts withholding aggressive care by years in practice. **B)** Chance of mortality that prompts withholding aggressive care by trauma center level. **C)** Chance of mortality that prompts withholding aggressive care by trauma volume per year. **D)** Chance of mortality that prompts withholding aggressive care by frequency of TBI care provided.

To explore factors associated with physician aggressiveness, we chose to use the mortality threshold at which physicians would consider withholding aggressive care as a surrogate measure of aggressiveness. We compared physician demographic values to the threshold value for mortality at which the physician would seriously consider withdrawing care. Years of physician experience did not significantly influence aggressiveness (p = 0.148, Fig 4A). Similarly, the physician’s trauma center designation, TBI volume, and personal frequency of TBI care did not significantly influence aggressiveness, although respondents at Level III trauma centers indicated they would withdraw care based on lower predicted mortality values than those at higher designated centers (p = 0.069, Fig 4B).

## Discussion

The development and validation of the IMPACT prognostic model was published in 2008[5]. The results of the univariate and multivariate prognostic analyses were published as a Supplemental Issue in the *Journal of Neurotrauma* in February 2007 [11]. Despite the many important achievements of the IMPACT study approximately a decade since its initial publication, relatively little is known about if and how clinicians are currently using the IMPACT prognostic calculator. It is our belief that the IMPACT prognostic calculator has been a substantial advancement in the care of TBI victims but that it is being insufficiently utilized. The IMPACT prognostic calculator could have particularly important utility in helping to reduce variability and nihilism in decisions surrounding care of the severely brain injured. The first step in achieving this possibility is to understand how providers are currently using the calculator and their views on the aggressiveness of patient care.

### Physician aggressiveness in the care of TBI patients

Despite the frequency with which most neurosurgeons make decisions about when to provide aggressive care to severely brain-injured patients, there has been little effort to reduce variability in these decisions or to objectify the notion of nihilism in this population. Although clinical judgement and individual patient characteristics must remain the most important factors in making these decisions, a better understanding of how prognostic calculations are interpreted by physicians may assist these decisions. We believe that there is great value in seeking consensus in what may constitute nihilism and that this may lead to the development of a safeguard against inappropriate therapeutic nihilism.

Neurotrauma physicians face a number of challenges early in the treatment course for brain-injured patients. These include but are not limited to communicating the nature of the insult as well as a sense of the prognosis that is as objective as possible, the time frame for neurological recovery, and the certainty in achieving the predicted functional state [12]. They must also establish a plan of care that reflects input from the family and the views of the medical team. Uncertainty inherent to the outcome that would be achieved with aggressive care adds greatly to the challenge of these tasks. Unfortunately, clinicians’ opinions vary greatly when it comes to outcome predictions [6, 13, 14] and tend to fall on the pessimistic side of the spectrum [6]. The IMPACT prognostic calculator can thus serve as an important and objective aid in the prognostication of moderate and severe TBI, although there has been no previous attempt to do this to our knowledge.

### The IMPACT prognostic calculator

Prediction of outcome after TBI has been a holy grail in medicine. A very large number of published papers describe prognostic variables in the severe TBI population and numerous prediction models have been described over the last century [5, 7, 15–45]. Accurate prediction of outcome in TBI patients can assist with counseling families, aid in determining appropriate resource utilization, and help to prevent undesirable outcomes. Indeed, academic efforts to define prognostic variables in TBI have been going on since at least 1923 [46].

The very large IMPACT database was developed in part to improve prognostic assessment of moderate and severe TBI victims. The IMPACT prognostic calculator is the most mature and studied prognostic calculator currently in use [8, 23, 47–52]. The large number of patients was important in overcoming the heterogeneity of the TBI population [53]. The IMPACT calculator (http://www.tbi-impact.org/?p=impact/calc) offers three highly validated [41] prognostic models (Core, Core + CT, and Core + CT + Lab) into which data available early after injury can be input [42]. The IMPACT prognostic calculator has been demonstrated to explain approximately 80% of the variability in patient outcomes, and its use is encouraged by the American College of Surgeons Trauma Quality Improvement Program as a means of scrutinizing the quality of care provided to TBI victims [54].

### Current use of the IMPACT prognostic calculator

Even though the IMPACT prognostic calculator has arguably been one of the most important advances in modern TBI care, the results of our survey suggest that physician awareness of this tool is insufficient among those who provide care to patients with moderate and severe TBI [25, 55]. Greater awareness among neurosurgeons was anticipated because the IMPACT model was predominantly a neurosurgical effort. Greater awareness and use among junior faculty likely reflects exposure to relevant IMPACT literature during training. Even among those that use the calculator, our survey indicates that it currently receives greatest use as a tool to improve communication with families; however, the IMPACT calculator has the potential to serve as a tool for much more.

Time needed to gather the data required to complete the online IMPACT calculation is likely an important factor limiting more extensive use and influence of the IMPACT calculator, especially in the most urgent clinical situations. This may underlie the reported lack of use and influence of the calculator at least to some extent. To address this problem our group has embedded the IMPACT calculator within a widely used medical record software, automating much of the data gathering and shortening the time to perform a calculation. This embedded calculator is a subject of ongoing work and investigation by our group.

### Consensus on when to provide aggressive care

According to the survey responses we received, there seems to be a strong consensus on when it may be reasonable to pursue comfort care. When including all responses, the average chance of mortality as predicted by the IMPACT calculator that would cause prompt pursuit of palliative care was 86.7%. This number was 85.9%—very similar—when participants were asked about chance of poor outcome and pursuit of palliative care. Our data demonstrate a strong consensus among physicians that a high chance of mortality and/or poor outcome is required before withdrawal of aggressive care is considered. Interestingly, the thresholds we identified align with the findings of a 2011 study looking at the cost-effectiveness of decompressive craniectomy for severe TBI [56], which suggested that the hospital costs associated with this procedure increase as the severity of TBI increases and peak at a predicted risk of unfavorable outcome around 80%.

Our survey also demonstrated robust agreement that aggressive care should be strongly considered in patients younger than an age threshold of 76 years. Age is the strongest predictor of outcome after TBI [57]; remarkably, its prognostic importance exceeds that of the Glasgow Coma Scale motor score. Interestingly, the age threshold identified in our study aligns with a 2012 study published by Whitmore et al. [58] which showed that, when all the costs of severe TBI are considered, aggressive treatment is a cost-effective option compared with “routine care” up to 80 years of age.

### Prognostic thresholds must not be misused

A very important contribution of IMPACT’s high-resolution, “big-data” analysis of the relationship between continuous variables and outcome was to dispel the notion of thresholds in TBI prognostication [59]. A spline function failed to suggest a threshold age at which there is a marked change in outcome, for instance. Thresholds in the TBI field have generally arisen as a result of arbitrary cut-points to dichotomize data for statistical analysis. Nonetheless, the notion of thresholds is entrenched in the care of TBI victims as guidelines recommend treatment thresholds for systolic blood pressure, intracranial pressure, cerebral perfusion pressure, and jugular venous oxygen saturation. This reflects the practicality that values must be identified at which treatment should be initiated to mitigate harm.

We feel strongly that the thresholds identified in our work should not be misused. Moreover, physicians must consider that these are consensus-based thresholds and that the age threshold we have identified is not associated with a marked change in outcome–outcome at the age of 77 is similar to that seen at the age of 75 for instance [59]. All decisions related to aggressiveness of care must be individualized, and the thresholds we have identified should not by themselves constitute reason for pursuing non-aggressive care. Here it is important to consider the misuse of an age threshold discussed in the first prognosis guidelines of the Brain Trauma Foundation, which were paired with the second edition of the Guidelines for the Management of Severe TBI [3]. An age threshold of 65 years was discussed in the Brain Trauma Foundation’s Prognostic Guidelines, and this prompted some to withhold treatment even for healthy individuals older than 65. Decisions restricting aggressive care in older individuals or those with chance of death or poor outcome exceeding 85% should have a strong basis in individual characteristics, family wishes, and physician judgement.

## Limitations

A number of limitations of our work must be considered. Surveys involving voluntary participation such as this may not reflect the opinions of non-respondents and may thus not represent true consensus. It is remarkable, however, that the thresholds identified in our work were consistent amongst key subgroups. It must also be considered that a survey of physician opinions such as this may not accurately reflect real-world behavior. While it would be interesting to compare physician opinion with physician practice such an investigation is beyond the scope of this survey. Comparing such stated opinion with real-world practice is an important subject for future research.

## Conclusions

This study shows that many physicians who provide care to patients with moderate or severe TBI are unaware of the IMPACT prognostic calculator. Of those clinicians that were aware of the calculator, most use the tool to aid discussion with families, and few reported that the prognostic calculations strongly influence their care of TBI patients. Our multidisciplinary, international survey provides valuable information as to the aggressiveness of physicians, and it identified robust thresholds for predicted poor outcome, mortality, and age which–with further study–may ultimately help to bring greater objectivity to level-of-care decisions for the most severely injured patients.

## Supporting information

S1 Survey Physician survey. (PDF)

## References

[1] Hawryluk GW, Bullock MR. Design of acute neuroprotection studies. Handb Clin Neurol. 2015;128:761–78. doi: 10.1016/B978-0-444-63521-1.00047-9 . 25701919 10.1016/B978-0-444-63521-1.00047-9

[2] Hawryluk GW, Bullock MR. Past, Present, and Future of Traumatic Brain Injury Research. Neurosurg Clin N Am. 2016;27(4):375–96. doi: 10.1016/j.nec.2016.05.002 . 27637391 10.1016/j.nec.2016.05.002

[3] Bullock R, Chesnut RM, Clifton G, Ghajar J, Marion DW, Narayan RK, et al Guidelines for the management of severe head injury. Brain Trauma Foundation. Eur J Emerg Med. 1996;3(2):109–27. . 9028756 10.1097/00063110-199606000-00010

[4] Shafi S, Barnes SA, Millar D, Sobrino J, Kudyakov R, Berryman C, et al Suboptimal compliance with evidence-based guidelines in patients with traumatic brain injuries. J Neurosurg. 2014;120(3):773–7. doi: 10.3171/2013.12.JNS132151 . 24438538 10.3171/2013.12.JNS132151

[5] Steyerberg EW, Mushkudiani N, Perel P, Butcher I, Lu J, McHugh GS, et al Predicting outcome after traumatic brain injury: development and international validation of prognostic scores based on admission characteristics. PLoS Med. 2008;5(8):e165; discussion e. doi: 10.1371/journal.pmed.0050165 ; PubMed Central PMCID: PMCPMC2494563. 18684008 10.1371/journal.pmed.0050165 PMC2494563

[6] Moore NA, Brennan PM, Baillie JK. Wide variation and systematic bias in expert clinicians' perceptions of prognosis following brain injury. Br J Neurosurg. 2013;27(3):340–3. doi: 10.3109/02688697.2012.754402 . 23461749 10.3109/02688697.2012.754402

[7] Castano-Leon AM, Lora D, Munarriz PM, Cepeda S, Paredes I, de la Cruz J, et al Predicting Outcomes after Severe and Moderate Traumatic Brain Injury: An External Validation of Impact and Crash Prognostic Models in a Large Spanish Cohort. J Neurotrauma. 2016;33(17):1598–606. doi: 10.1089/neu.2015.4182 . 26982260 10.1089/neu.2015.4182

[8] Roozenbeek B, Chiu YL, Lingsma HF, Gerber LM, Steyerberg EW, Ghajar J, et al Predicting 14-day mortality after severe traumatic brain injury: application of the IMPACT models in the brain trauma foundation TBI-trac(R) New York State database. J Neurotrauma. 2012;29(7):1306–12. doi: 10.1089/neu.2011.1988 ; PubMed Central PMCID: PMCPMC3335134. 22150207 10.1089/neu.2011.1988 PMC3335134

[9] Tasaki O, Shiozaki T, Hamasaki T, Kajino K, Nakae H, Tanaka H, et al Prognostic indicators and outcome prediction model for severe traumatic brain injury. J Trauma. 2009;66(2):304–8. doi: 10.1097/TA.0b013e31815d9d3f . 19204501 10.1097/TA.0b013e31815d9d3f

[10] Harris PA, Taylor R, Thielke R, Payne J, Gonzalez N, Conde JG. Research electronic data capture (REDCap)—a metadata-driven methodology and workflow process for providing translational research informatics support. J Biomed Inform. 2009;42(2):377–81. doi: 10.1016/j.jbi.2008.08.010 ; PubMed Central PMCID: PMCPMC2700030. 18929686 10.1016/j.jbi.2008.08.010 PMC2700030

[11] Marmarou A, Lu J, Butcher I, McHugh GS, Mushkudiani NA, Murray GD, et al IMPACT database of traumatic brain injury: design and description. J Neurotrauma. 2007;24(2):239–50. doi: 10.1089/neu.2006.0036 . 17375988 10.1089/neu.2006.0036

[12] Kirschen MP, Walter JK. Ethical Issues in Neuroprognostication after Severe Pediatric Brain Injury. Semin Pediatr Neurol. 2015;22(3):187–95. doi: 10.1016/j.spen.2015.05.004 . 26358429 10.1016/j.spen.2015.05.004

[13] Kaufmann MA, Buchmann B, Scheidegger D, Gratzl O, Radu EW. Severe head injury: should expected outcome influence resuscitation and first-day decisions? Resuscitation. 1992;23(3):199–206. . 1321479 10.1016/0300-9572(92)90003-u

[14] Turgeon AF, Lauzier F, Burns KE, Meade MO, Scales DC, Zarychanski R, et al Determination of neurologic prognosis and clinical decision making in adult patients with severe traumatic brain injury: a survey of Canadian intensivists, neurosurgeons, and neurologists. Crit Care Med. 2013;41(4):1086–93. doi: 10.1097/CCM.0b013e318275d046 . 23385104 10.1097/CCM.0b013e318275d046

[15] Baltazar GA, Pate AJ, Panigrahi B, LaBoy S, Prosniak R, Mody A, et al Malnutrition as measured by albumin and prealbumin on admission is associated with poor outcomes after severe traumatic brain injury. Am Surg. 2015;81(2):E61–3. . 25642858

[16] Bazzazi A, Hasanloei MA, Mahoori A, Gholamnejad M, Tarverdipour H. Correlation between arterial blood gas analysis and outcome in patients with severe head trauma. Ulus Travma Acil Cerrahi Derg. 2014;20(4):236–40. doi: 10.5505/tjtes.2014.57089 . 25135016 10.5505/tjtes.2014.57089

[17] Brain Trauma Foundation, American Association of Neurological Surgeons, Congress of Neurological Surgeons. Guidelines for the management of severe traumatic brain injury. J Neurotrauma. 2007;24 Suppl 1:S1–106. doi: 10.1089/neu.2007.9999 . 17511534 10.1089/neu.2007.9999

[18] Brandner S, Kellermann I, Hore N, Bozhkov Y, Buchfelder M. Clinical Course Score (CCS): a new clinical score to evaluate efficacy of neurotrauma treatment in traumatic brain injury and subarachnoid hemorrhage. J Neurosurg Anesthesiol. 2015;27(1):26–30. doi: 10.1097/ANA.0000000000000083 . 24879534 10.1097/ANA.0000000000000083

[19] Carter EL, Hutchinson PJ, Kolias AG, Menon DK. Predicting the outcome for individual patients with traumatic brain injury: a case-based review. Br J Neurosurg. 2016;30(2):227–32. doi: 10.3109/02688697.2016.1139048 . 26853860 10.3109/02688697.2016.1139048

[20] Czeiter E, Mondello S, Kovacs N, Sandor J, Gabrielli A, Schmid K, et al Brain injury biomarkers may improve the predictive power of the IMPACT outcome calculator. J Neurotrauma. 2012;29(9):1770–8. doi: 10.1089/neu.2011.2127 ; PubMed Central PMCID: PMCPMC3409455. 22435839 10.1089/neu.2011.2127 PMC3409455

[21] Gao J, Zheng Z. Development of prognostic models for patients with traumatic brain injury: a systematic review. Int J Clin Exp Med. 2015;8(11):19881–5. ; PubMed Central PMCID: PMCPMC4723744. 26884899 PMC4723744

[22] Gao L, Wu X. Prediction of clinical outcome in severe traumatic brain injury. Front Biosci (Landmark Ed). 2015;20:763–71. . 25553477 10.2741/4335

[23] Greene NH, Kernic MA, Vavilala MS, Rivara FP. Variation in pediatric traumatic brain injury outcomes in the United States. Arch Phys Med Rehabil. 2014;95(6):1148–55. doi: 10.1016/j.apmr.2014.02.020 ; PubMed Central PMCID: PMCPMC4146619. 24631594 10.1016/j.apmr.2014.02.020 PMC4146619

[24] Harrison DA, Prabhu G, Grieve R, Harvey SE, Sadique MZ, Gomes M, et al Risk Adjustment In Neurocritical care (RAIN)—prospective validation of risk prediction models for adult patients with acute traumatic brain injury to use to evaluate the optimum location and comparative costs of neurocritical care: a cohort study. Health Technol Assess. 2013;17(23):vii–viii, 1–350. doi: 10.3310/hta17230 ; PubMed Central PMCID: PMCPMC4781166. 23763763 10.3310/hta17230 PMC4781166

[25] Iorio-Morin C, Fortin D, Blanchard J. TBI prognosis calculator: A mobile application to estimate mortality and morbidity following traumatic brain injury. Clin Neurol Neurosurg. 2016;142:48–53. doi: 10.1016/j.clineuro.2016.01.021 . 26808078 10.1016/j.clineuro.2016.01.021

[26] Kamal VK, Agrawal D, Pandey RM. Prognostic models for prediction of outcomes after traumatic brain injury based on patients admission characteristics. Brain Inj. 2016;30(4):393–406. doi: 10.3109/02699052.2015.1113568 . 27003280 10.3109/02699052.2015.1113568

[27] Kawai N, Kawanishi M, Kudomi N, Maeda Y, Yamamoto Y, Nishiyama Y, et al Detection of brain amyloid beta deposition in patients with neuropsychological impairment after traumatic brain injury: PET evaluation using Pittsburgh Compound-B. Brain Inj. 2013;27(9):1026–31. doi: 10.3109/02699052.2013.794963 . 23662609 10.3109/02699052.2013.794963

[28] Kumar RG, Boles JA, Wagner AK. Chronic Inflammation After Severe Traumatic Brain Injury: Characterization and Associations With Outcome at 6 and 12 Months Postinjury. J Head Trauma Rehabil. 2015;30(6):369–81. doi: 10.1097/HTR.0000000000000067 . 24901329 10.1097/HTR.0000000000000067

[29] Lazaridis C, DeSantis SM, Smielewski P, Menon DK, Hutchinson P, Pickard JD, et al Patient-specific thresholds of intracranial pressure in severe traumatic brain injury. J Neurosurg. 2014;120(4):893–900. doi: 10.3171/2014.1.JNS131292 . 24506248 10.3171/2014.1.JNS131292

[30] Liedes H, Mattila J, Lingsma H, Lotjonen J, Menon D, Tenovuo O, et al Prediction of Outcome after Traumatic Brain Injury: Comparison of Disease State Index and IMPACT Calculator. Stud Health Technol Inform. 2016;224:175–80. . 27225575

[31] Lu J, Roe C, Aas E, Lapane KL, Niemeier J, Arango-Lasprilla JC, et al Traumatic brain injury: methodological approaches to estimate health and economic outcomes. J Neurotrauma. 2013;30(23):1925–33. doi: 10.1089/neu.2013.2891 . 23879599 10.1089/neu.2013.2891

[32] Maas AI, Lingsma HF, Roozenbeek B. Predicting outcome after traumatic brain injury. Handb Clin Neurol. 2015;128:455–74. doi: 10.1016/B978-0-444-63521-1.00029-7 . 25701901 10.1016/B978-0-444-63521-1.00029-7

[33] Majdan M, Lingsma HF, Nieboer D, Mauritz W, Rusnak M, Steyerberg EW. Performance of IMPACT, CRASH and Nijmegen models in predicting six month outcome of patients with severe or moderate TBI: an external validation study. Scand J Trauma Resusc Emerg Med. 2014;22:68 doi: 10.1186/s13049-014-0068-9 ; PubMed Central PMCID: PMCPMC4267426. 25406964 10.1186/s13049-014-0068-9 PMC4267426

[34] Mikola A, Ratsep I, Sarkela M, Lipping T. Prediction of outcome in traumatic brain injury patients using long-term qEEG features. Conf Proc IEEE Eng Med Biol Soc. 2015;2015:1532–5. doi: 10.1109/EMBC.2015.7318663 . 26736563 10.1109/EMBC.2015.7318663

[35] Perrin PB, Niemeier JP, Mougeot JL, Vannoy CH, Hirsch MA, Watts JA, et al Measures of injury severity and prediction of acute traumatic brain injury outcomes. J Head Trauma Rehabil. 2015;30(2):136–42. doi: 10.1097/HTR.0000000000000026 . 24590151 10.1097/HTR.0000000000000026

[36] Pourahmad S, Hafizi-Rastani I, Khalili H, Paydar S. Identifying Important Attributes for Prognostic Prediction in Traumatic Brain Injury Patients. A Hybrid Method of Decision Tree and Neural Network. Methods Inf Med. 2016;55(5):440–9. doi: 10.3414/ME15-01-0080 . 27492342 10.3414/ME15-01-0080

[37] Raj R, Siironen J, Kivisaari R, Hernesniemi J, Skrifvars MB. Predicting outcome after traumatic brain injury: development of prognostic scores based on the IMPACT and the APACHE II. J Neurotrauma. 2014;31(20):1721–32. doi: 10.1089/neu.2014.3361 ; PubMed Central PMCID: PMCPMC4179932. 24836936 10.1089/neu.2014.3361 PMC4179932

[38] Ramesh VG. Outcome prediction in traumatic brain injury. J Neurosurg. 2013;119(5):1351 doi: 10.3171/2013.4.JNS13875 . 23952036 10.3171/2013.4.JNS13875

[39] Rizoli S, Petersen A, Bulger E, Coimbra R, Kerby JD, Minei J, et al Early prediction of outcome after severe traumatic brain injury: a simple and practical model. BMC Emerg Med. 2016;16(1):32 doi: 10.1186/s12873-016-0098-x ; PubMed Central PMCID: PMCPMC4995825. 27553436 10.1186/s12873-016-0098-x PMC4995825

[40] Shaklai S, Peretz R, Spasser R, Simantov M, Groswasser Z. Long-term functional outcome after moderate-to-severe paediatric traumatic brain injury. Brain Inj. 2014;28(7):915–21. doi: 10.3109/02699052.2013.862739 . 24826955 10.3109/02699052.2013.862739

[41] Sobuwa S, Hartzenberg HB, Geduld H, Uys C. Predicting outcome in severe traumatic brain injury using a simple prognostic model. S Afr Med J. 2014;104(7):492–4. doi: 10.7196/samj.7720 . 25214051 10.7196/samj.7720

[42] Sun H, Lingsma HF, Steyerberg EW, Maas AI. External Validation of the International Mission for Prognosis and Analysis of Clinical Trials in Traumatic Brain Injury: Prognostic Models for Traumatic Brain Injury on the Study of the Neuroprotective Activity of Progesterone in Severe Traumatic Brain Injuries Trial. J Neurotrauma. 2016;33(16):1535–43. doi: 10.1089/neu.2015.4164 . 26652051 10.1089/neu.2015.4164

[43] Talari HR, Fakharian E, Mousavi N, Abedzadeh-Kalahroudi M, Akbari H, Zoghi S. The Rotterdam Scoring System Can Be Used as an Independent Factor for Predicting Traumatic Brain Injury Outcomes. World Neurosurg. 2016;87:195–9. doi: 10.1016/j.wneu.2015.11.055 . 26704195 10.1016/j.wneu.2015.11.055

[44] Wong GK, Teoh J, Yeung J, Chan E, Siu E, Woo P, et al Outcomes of traumatic brain injury in Hong Kong: validation with the TRISS, CRASH, and IMPACT models. J Clin Neurosci. 2013;20(12):1693–6. doi: 10.1016/j.jocn.2012.12.032 . 23993210 10.1016/j.jocn.2012.12.032

[45] Zador Z, Sperrin M, King AT. Predictors of Outcome in Traumatic Brain Injury: New Insight Using Receiver Operating Curve Indices and Bayesian Network Analysis. PLoS One. 2016;11(7):e0158762 doi: 10.1371/journal.pone.0158762 ; PubMed Central PMCID: PMCPMC4936732. 27388421 10.1371/journal.pone.0158762 PMC4936732

[46] Butler E. Brain Injuries, Mechanics, Prognosis, and Treatment. Cal State J Med. 1923;21(7):295–6. ; PubMed Central PMCID: PMCPMC1517637. 18739043 PMC1517637

[47] Lingsma H, Andriessen TM, Haitsema I, Horn J, van der Naalt J, Franschman G, et al Prognosis in moderate and severe traumatic brain injury: external validation of the IMPACT models and the role of extracranial injuries. J Trauma Acute Care Surg. 2013;74(2):639–46. doi: 10.1097/TA.0b013e31827d602e . 23354263 10.1097/TA.0b013e31827d602e

[48] Lingsma HF, Roozenbeek B, Li B, Lu J, Weir J, Butcher I, et al Large between-center differences in outcome after moderate and severe traumatic brain injury in the international mission on prognosis and clinical trial design in traumatic brain injury (IMPACT) study. Neurosurgery. 2011;68(3):601–7; discussion 7–8. doi: 10.1227/NEU.0b013e318209333b . 21311293 10.1227/NEU.0b013e318209333b

[49] Olivecrona M, Koskinen LO. The IMPACT prognosis calculator used in patients with severe traumatic brain injury treated with an ICP-targeted therapy. Acta Neurochir (Wien). 2012;154(9):1567–73. doi: 10.1007/s00701-012-1351-z . 22543506 10.1007/s00701-012-1351-z

[50] Panczykowski DM, Puccio AM, Scruggs BJ, Bauer JS, Hricik AJ, Beers SR, et al Prospective independent validation of IMPACT modeling as a prognostic tool in severe traumatic brain injury. J Neurotrauma. 2012;29(1):47–52. doi: 10.1089/neu.2010.1482 . 21933014 10.1089/neu.2010.1482

[51] Roozenbeek B, Lingsma HF, Lecky FE, Lu J, Weir J, Butcher I, et al Prediction of outcome after moderate and severe traumatic brain injury: external validation of the International Mission on Prognosis and Analysis of Clinical Trials (IMPACT) and Corticoid Randomisation After Significant Head injury (CRASH) prognostic models. Crit Care Med. 2012;40(5):1609–17. doi: 10.1097/CCM.0b013e31824519ce ; PubMed Central PMCID: PMCPMC3335746. 22511138 10.1097/CCM.0b013e31824519ce PMC3335746

[52] Yeoman P, Pattani H, Silcocks P, Owen V, Fuller G. Validation of the IMPACT outcome prediction score using the Nottingham Head Injury Register dataset. J Trauma. 2011;71(2):387–92. doi: 10.1097/TA.0b013e31820ceadd . 21427619 10.1097/TA.0b013e31820ceadd

[53] Maas AI, Murray GD, Roozenbeek B, Lingsma HF, Butcher I, McHugh GS, et al Advancing care for traumatic brain injury: findings from the IMPACT studies and perspectives on future research. Lancet Neurol. 2013;12(12):1200–10. doi: 10.1016/S1474-4422(13)70234-5 ; PubMed Central PMCID: PMCPMC3895622. 24139680 10.1016/S1474-4422(13)70234-5 PMC3895622

[54] Carney N, Totten AM, O'Reilly C, Ullman JS, Hawryluk GW, Bell MJ, et al Guidelines for the Management of Severe Traumatic Brain Injury, Fourth Edition. Neurosurgery. 2016 doi: 10.1227/NEU.0000000000001432 . 27654000 10.1227/NEU.0000000000001432

[55] Menon DK, Zahed C. Prediction of outcome in severe traumatic brain injury. Curr Opin Crit Care. 2009;15(5):437–41. doi: 10.1097/MCC.0b013e3283307a26 . 19713837 10.1097/MCC.0b013e3283307a26

[56] Alvis-Miranda H, Castellar-Leones SM, Moscote-Salazar LR. Decompressive Craniectomy and Traumatic Brain Injury: A Review. Bull Emerg Trauma. 2013;1(2):60–8. ; PubMed Central PMCID: PMCPMC4771225. 27162826 PMC4771225

[57] Murray GD, Butcher I, McHugh GS, Lu J, Mushkudiani NA, Maas AI, et al Multivariable prognostic analysis in traumatic brain injury: results from the IMPACT study. J Neurotrauma. 2007;24(2):329–37. doi: 10.1089/neu.2006.0035 . 17375997 10.1089/neu.2006.0035

[58] Whitmore RG, Thawani JP, Grady MS, Levine JM, Sanborn MR, Stein SC. Is aggressive treatment of traumatic brain injury cost-effective? J Neurosurg. 2012;116(5):1106–13. doi: 10.3171/2012.1.JNS11962 . 22394292 10.3171/2012.1.JNS11962

[59] Mushkudiani NA, Engel DC, Steyerberg EW, Butcher I, Lu J, Marmarou A, et al Prognostic value of demographic characteristics in traumatic brain injury: results from the IMPACT study. J Neurotrauma. 2007;24(2):259–69. doi: 10.1089/neu.2006.0028 . 17375990 10.1089/neu.2006.0028

