# Supplementary material for: The aggressiveness of neurotrauma practitioners and the influence of the IMPACT prognostic calculator
Source: PLoS One. 2017 Aug 23;12(8):e0183552. doi: 10.1371/journal.pone.0183552 (PMC5568296; doi:10.1371/journal.pone.0183552)
Supplement: S1 Survey — (PDF) [file pone.0183552.s001.pdf]

Physician Survey

Thank you for participating in this survey regarding IMPACT and the management of TBI patients. Please answer the questions below to the best of your knowledge.

Your responses will remain anonymous. Identifying information will not be included in any publication that may arise from this survey.

At what institution do you practice? ______________________________

How many years have you been in practice? ______________________________

In what medical specialty did you complete your residency? ______________________________

If you completed sub-specialty training please report the specific field here. Please leave blank if you do not have sub-specialty training. ______________________________

What level of trauma care does your trauma center provide?

- ◯ level I
- ◯ level II
- ◯ level III

What is the approximate volume of severe TBI seen at your hospital per year?

- ◯ 0-5
- ◯ 5-15
- ◯ 15-30
- ◯ 30-50
- ◯ 50-100
- ◯ 100-200
- ◯ 200+

(number of patients)

What is the approximate volume of moderate TBI seen at your hospital per year?

- ◯ 0-5
- ◯ 5-15
- ◯ 15-30
- ◯ 30-50
- ◯ 50-100
- ◯ 100-200
- ◯ 200+

(number of patients)

How often do you personally provide care to patients hospitalized for moderate or severe traumatic brain injury?

- ◯ Very frequently
- ◯ Frequently
- ◯ Less Frequently
- ◯ Occasionally
- ◯ Rarely
- ◯ Never

In your hospital, are your surgical and neurological intensive care units combined or separate?

- ◯ Combined
- ◯ Separate

To what degree does your institution follow the published Brain Trauma Foundation Guidelines?

- ◯ Always
- ◯ Most of the time
- ◯ Some of the time
- ◯ We don't make an effort to follow them

Does your institution use intraventricular catheters or intraparenchymal strain gauge technology for ICP monitoring?

- ◯ Mostly intraventricular catheters
- ◯ Mostly intraparenchymal monitors
- ◯ Either without clear preference

The IMPACT Prognostic Calculator (www.tbi-impact.org) provides predictions of death and poor outcome based on characteristics present on admission.

Are you aware of the IMPACT Prognostic Calculator which is available online?
- ◯ Yes
- ◯ No

Do you use the IMPACT Prognostic Calculator in the management of your brain injured patients?
- ◯ Never
- ◯ Sometimes
- ◯ Often

How much does the IMPACT Prognostic Calculator influence or alter your care of patients?
- ◯ No influence
- ◯ Mild influence
- ◯ Moderate influence
- ◯ Significant influence

In what ways does the IMPACT Prognostic Calculator influence your practice?
- ☐ Proceed more quickly to a lower level of care (withdrawal or palliation)
- ☐ Provide more aggressive care than you otherwise would
- ☐ Improve communication about prognosis with family
- ☐ Other

(Check all that apply)

If "Other" please specify ______________________________

The IMPACT investigators suggest that IMPACT's prognostic predictions should be applied to individual patients with caution. If the model produced predictions that were 100% reliable...

...What chance of mortality would cause you to pursue prompt withdrawal of care or comfort care?

0% 50% 100%

(Place a mark on the scale above)

...What chance of poor outcome would cause you to pursue prompt withdrawal of care or comfort care?

0% 50% 100%

(Place a mark on the scale above)

To what extent does patient age influence your decision to pursue aggressive management after traumatic brain injury?

None Moderate Substantial

(Place a mark on the scale above)

At what specific age do you seriously consider withholding aggressive TBI care. Please answer '0' if age does not cause you to seriously consider withholding aggressive TBI care.

0 100

(Place a mark on the scale above)
